# Haematological abnormalities in children with sickle cell disease and non-severe malaria infection in western Kenya

**DOI:** 10.1186/s12879-021-06025-7

**Published:** 2021-04-07

**Authors:** Paul Kosiyo, Walter Otieno, Jesse Gitaka, Elly O. Munde, Collins Ouma

**Affiliations:** 1 Department of Biomedical Science and Technology, School of Public Health and Community Development, Maseno University, Private Bag, Maseno, Kenya; 2 Department of Medical Laboratory Sciences, School of Medicine, Maseno University, Private Bag, Maseno, Kenya; 3 Department of Paediatrics and Child Health, School of Medicine, Maseno University, Private Bag, Maseno, Kenya; 4 Directorate of Research and Innovation, Mount Kenya University, General Kago Road, P.O. Box 342-01000, Thika, Kenya; 5 Department of Clinical Medicine, School of Health Sciences, Kirinyaga University, P.O. Box 143-10300, Kerugoya, Kenya

**Keywords:** *HBB*, Genotype, Haematological parameter, *P. falciparum*

## Abstract

**Background:**

In *Plasmodium falciparum* infection, clinical conditions such as anaemia, thrombocytopenia and leukocytosis are common. Mutation in haemoglobin sub-unit beta gene (*HBB*) may be a genetic factor responsible for these haematological changes during infection. However, the contributions of the carriage of different *HBB* genotypes on these changes remain largely unknown.

**Methodology:**

In this cross-sectional study, we evaluated haematological abnormalities in *P. falciparum*-infected children (*n* = 217, aged 1–192 months) with different haemoglobin sub-unit beta (*HBB*) genotypes (HbAA, HbAS and HbSS). Children with acute febrile conditions were recruited at Jaramogi Oginga Odinga Teaching and Referral Hospital at the outpatient clinic. Haematological parameters were determined using Beckman Coulter counter ACTdiff2™ while *HBB* genotyping was done using TaqMan® SNP genotyping assay. Chi-square (χ^2^) was used to determine differences between proportions. Differences in haematological parameters were compared across groups using Kruskal Wallis test and between groups using Mann Whitney U test. Partial correlation test was used to determine correlation between haematological parameters and sickle cell genotypes while controlling for age and sex.

**Results:**

Haemoglobin (Hb), [median (IQR); 7.3 (1.3), *P =* 0.001], haematocrit (HCT), [median (IQR); 26.4 (4.4), *P =* 0.009], red blood cells (RBC), [median (IQR); 3.2 (1.7), *P =* 0.048] were markedly reduced in HbSS, however, red cell distribution with (RDW) [median (IQR); 14.9 (3.3), *P =* 0.030] was increased in malaria infected children with HbSS. Severe anaemia was highest in HbSS (23.1%) followed by HbAA (8.6%) and HbAS (7.1%). There were no differences in platelet count (*P* = 0.399) hence no severe thrombocytopeania across the genotypes. Leukocytosis was highest in HbSS (69.2%), 42% in HbAS and 31% in HbAA. The RBC, HCT and Hb had negative correlation with RDW in HbSS in malarial-infected children (*r* = − 0.725, *P* = 0.008), (*r* = − 0.718, *P* = 0.009) and (*r* = − 0.792, *P* = 0.002), respectively.

**Conclusion:**

Our study reveals that anaemia is the most common abnormality in malaria-infected children with carriage of HbSS. The RBC, HCT and Hb concentration decrease with increase in RDW levels in infected children with carriage of HbSS compared to other *HBB* genotypes. Therefore, carriage of HbSS genotype is correlated with severity of haematological abnormalities.

**Supplementary Information:**

The online version contains supplementary material available at 10.1186/s12879-021-06025-7.

## Background

One of the most important parasitic disease of man is malaria and it is known to claim more lives of children worldwide compared to other infectious diseases [1]. According to WHO estimates, Africa contributes up to 91% of malaria deaths worldwide [2]. Between the year 2000 and 2017, a significant decline in malaria incidence was achieved [2]. Kisumu County in western Kenya is one of the malaria holoendemic regions in Kenya with malaria prevalence at 27% [3]. In malaria holoendemic zones, *P. falciparum* is the most deadly and most prevalent species [4]. Severity of malaria varies from person to person and this variation is attributed to both host and parasite factors [5]. *P. falciparum* malaria severity is characterized by overlapping clinical presentations that include severe malaria anaemia (SMA), respiratory distress, metabolic acidosis, cerebral malaria (CM) and hypoglycaemia [6]. In western Kenya, severe malaria manifest as severe anaemia with Hb < 6.0 g/dL and any density parasitaemia in children below 5 years [7]. Furthermore, several studies in Africa especially in Nigeria [8] and Kenya [9] demonstrated that platelet count is significantly reduced in severe malaria infection and thus recommended the inclusion of thrombocytopenia in case definition of severe malaria. The latter study which was equally conducted in the current study site further determined a correlation between anaemia and thrombocytopenia in *P. falciparum* infected children with normal haemoglobin (HbAA) [9]. However, the above study did not consider different haemoglobin beta sub-unit (*HBB*) genotypes more so sickle cell gene mutation (6G**A**G > 6G**T**G). Moreover, as shown by several studies in western Kenya, it is plausible that SMA in children is as a result of imbalance in the production of both pro-inflammatory and anti-inflammatory mediators, polymorphisms in immune regulatory genes and exacerbations in the presence of co-infections with HIV-1 and pathogenic bacteria [10–12]. Whether malaria is severe or non-severe, it is important to note that the overlapping interactions between haemoglobinopathy especially in the haemoglobin beta sub-unit gene (*HBB*) and malaria, remains a complex biological conundrum that is not well understood [13].

In certain individuals, *HBB* may contain mutation for the sickle cell gene (6G**A**G > 6G**T**G). The *HBB* mutation (6G**A**G > 6G**T**G) is due to nucleotide substitution of thymine for adenine at the sixth codon of *HBB*. This results in incorporation of valine, instead of glutamine culminating into haemoglobin tetramers (HbS) that aggregate into arrays when deoxygenated in the tissues [14]. The global effect of sickle cell disease (SCD) has been projected to be approximately 275,000 deliveries per annum [15] and it is also approximated that it could reach 400,000 childbirths by 2050 in repute to recent projections. The geographical distribution of these SCD is very similar to that of malaria and it has been found that SCT confers some resistance to malaria giving a chance to malaria sickle cell disease interface [15]. The *P. falciparum* infection is habitually fatal in individuals with sickle cell anaemia (HbSS) since the protection from infection seems to operate in HbS dose-dependent manner thus individuals with HbSS have an even reduced risk of infection than those with HbAS [16].

Even though sickle cell disease (SCD) is primarily a disease of red blood cells, both leukocytes and thrombocytes are equally affected just like in malaria infection and these are known to drive sickle cell crises via vaso-occlusion [17]. This has creating a need to further understand the severities in the context of haematological abnormalities. Additionally, monocytosis has been found to be associated with haemolysis and inflammation in sickle cell anaemia [18]. However, the influence of the carriage of different *HBB* genotypes (6G**A**G > 6G**T**G) on the severities of major haematological abnormalities and correlation of haematological parameters with *HBB* genotypes (6G**A**G > 6G**T**G) once children are infected with *P. falciparum* malaria, remains unknown. As such, we determined the severity of haematological abnormalities and correlation of haematological parameters with different haemoglobin beta sub-unit (*HBB*) genotypes in children with *P. falciparum* malaria in Kisumu County, western Kenya.

## Materials and methods

### Study site

Details on the current study site has been previously described in our publication [19]. In brief, the study was conducted at Jaramogi Oginga Odinga Teaching and Referral Hospital (JOOTRH) in Kisumu County between April 2018 and February 2019. JOOTRH is a major referral hospital in western Kenya and it is located within the headquarters of Kisumu County. Kisumu County is a malaria endemic area with prevalence at 27% [20].

### Study population

This was a cross-sectional study targeting children aged 1–192 months and resident within Kisumu County (See Supplementary Data File I). The sample size was calculated based on prevalence of sickle cell trait in the study area [21] using Cochran’s formula [22]. The details of the study population are described in details in our previous publication [19].

### Inclusion criteria

Children found to be infected with *P. falciparum* upon demonstration of asexual forms of *P. falciparum* through microscopic examination of both thick and thin smear, children whose parents were willing and able to provide written informed consent were included in the study.

### Exclusion criteria

Children with previously known forms of haemoglobinopathies e.g. α-thalassaemia syndromes, history of sickle cell crises and blood transfusion in the past 3 months, acute bacterial, viral infections and parasitic infections other than *P. falciparum*, and any form of malignancy and known sickle cell patients on hydroxyurea therapy.

### Ethical consideration

The study was approved by Jaramogi Oginga Odinga Teaching and Referral Hospital Scientific and Ethics Review Committee (JOOTRH-ERC) - Approval NO. ERC.IB/VOL.1/414. All methods were performed in accordance with the relevant guidelines and regulations.

### Laboratory procedures

The details of the laboratory procedures such as haematological measurements, microscopy for demonstration of any asexual form of *P. falciparum*, DNA extraction and quantification are described in details in our previous publication [19]. In summary, haematological measurements were determined using Beckman Coulter counter ACTdiff2™, *P. falciparum* infection status was determined by microscopy. PureLink® DNA Mini Kit (Invitrogen life technologies, USA) was used for DNA extraction while DNA quality and quantity was assessed using Nano Drop ND-1000 spectrophotometer (Thermofisher Scientific, San Diego, CO, USA) and stored at -20 °C prior to use.

### *HBB* rs334 genotyping

Genotyping of the *HBB* rs334 SNP was performed using TaqMan® SNP Genotyping Assay in accordance with manufacturer’s protocols (Life Technologies, Grand Island, NY). Identification of haemoglobin S was from biallellic discrimination (missense change) [Glu6VAL] in the single nucleotide polymorphism rs334 by the following custom primer and probe sequences: Forward-TCAAACAGACACCATGGTGCAT, Reverse-CCCCACAGGGCAGTAACG, VIC-CTGACTCCTGAGGAGAA-MGB, 6FAM-CTGACTCCTGTGGAGAA-MGB, respectively and as per our previous work [19]. For quality assurance, a triplicate of control samples obtained from our laboratory archived positive samples for genotype HbAA, HbAS and HbSS were also run. Amplification was performed in Real-time PCR StepOnePlus thermocycler from Applied Biosystems® (Foster City, CA, USA) through an initial denaturation at 95^o^ C for 10 min, followed by 40 cycles of denaturation at 95 °C for 1 min, annealing at 60^o^ C for 1 min and final extension at 72 °C for 1 min.

### Statistical analyses

Statistical analyses were performed using Statistical Package for the Social Sciences (SPSS) version 22.0 software (IBM, New York, USA). Chi-square (χ^2^) analysis was used to determine differences between proportions. Mann-Whitney U test and Kruskal Wallis test were used for comparisons of demographic and laboratory characteristics wherever applicable. Further post-hoc pair-wise analysis using Dunn’s multiple comparison test was used where there was statistical significance. Partial correlation test was used to determine the correlation between primary red cell measurements and red cell indices in children with carriage of *HBB* mutation (6G**A**G > 6G**T**G) genotypes while controlling for age and sex as confounders. *HBB* rs334 allele frequencies, consistency and/or deviations from Hardy-Weinberg Equilibrium (HWE) were determined using web-based site http://www.dr-petrek.eu/documents/HWE.xls. Statistical significance was set at *P* ≤ 0.05.

## Results

### Demographic characteristics of study participants

Children (*n* = 217, aged 1–192 months) with acute febrile conditions (temperature > 37.5 °C) were enrolled in the study. The participants were broadly categorized based on malaria infection status upon microscopic examination and demonstration of any asexual form of *P. falciparum* malaria (trophozoite or schizont) as non-infected (*n* = 132) and infected (*n* = 85). Sex (*P* = 0.240) and age (*P* = 0.143) were comparable between the two groups.

### *HBB* genotype distribution

Distribution of *HBB* rs334 genotypes in the study participants was also determined. Among the malaria negative patients, 90 (68.2%) had HbAA, 31 (23.5%) had HbAS and 11 (8.3%) had HbSS genotypes while in the infected group, 58 (68.2%) has HbAA, 14 (16.5%) had HbAS and 13 (15.3%) had HbSS genotype. Generally, haemoglobin types were comparable with regard to *P. falciparum* infection, *P* = 0.743. The overall genotype distribution for the *HBB* rs334 deviated significantly from the HWE (χ^2^ = 32.02, *P* < 0.001) with a minor allele frequency of 0.21.

### Haematological characteristics of all study participants

Haematological markers for anaemia i.e. haemoglobin, haematocrit and RBC count in those without *P. falciparum* parasitaemia were higher than those infected with *P. falciparum* malaria [(median (IQR), 10.5 (2.3) and 10.1 (3.2), *P =* 0.022], [(median (IQR), 35.2 (6.8) and 32.8 (9.0), *P =* 0.009] and [(median (IQR), 4.8 (0.7) and 4.6 (1.3), *P =* 0.045], respectively. Red cell distribution width (RDW, *P* = 0.703), Mean Corpuscular Volume (MCV, *P* = 0.349), Mean Corpuscular Heamoglobin (MCH, *P* = 0.744), Mean Corpuscular Haemoglobin Concentration (MCHC, *P* = 0.479), white blood cells (WBC, *P* = 0.746), lymphocytes (*P* = 0.103), monocytes (*P* = 0.084), and granulocytes (*P* = 0.354) counts were comparable between the two groups (Table 1). Further analysis revealed that the platelet counts were lower in the infected group [median (IQR), 236 (129.5) vs. non-infected group [median (IQR), 278 (112.8), *P* = 0.001, respectively. The mean platelet volume (MPV) were however, not different between the groups (*P* = 0.119). The children with malaria infection further had reduced plateletcrit (PCT), infected [median (IQR), 0.13 (0.1) vs. non-infected [median (IQR), 0.2 (0.1), *P* = 0.001. The platelet distribution width (PDW) were, however, comparable between the groups (*P* = 0.629).

**Table 1:** General Demographic and Laboratory Characteristics of the Study Participants

All the study participants (Infected and non-infected)				***P. falciparum*** infected children based on genotype (***n*** = 85)			
Characteristics	Non-Infected (***n*** = 132)	Infected (***n*** = 85)	***P-***value	HbAA (***n*** = 58)	HBAS (***n*** = 14)	HbSS (***n*** = 13)	***P-***Value
Sex, n (%)							
Male	76 (57.6)	42 (49.4)	0.240^a^	30 (51.7)	7 (50)	8 (61.5)	0.690^a^
Female	56 (42.4)	43 (50.6)		28 (48.3)	7 (50)	5 (38.5)	
Age, (months)	30 (48)	36 (55)	0.143^b^	36 (64.5)	36 (32.3)	16 (27)	0.660^c^
Haemoglobin type							
HbAA, n (%)	90 (68.2)	58 (68.2)					
HbAS, n (%)	31 (23.5)	14 (16.5)	0.743^a^				
HbSS, n (%)	11 (8.3)	13 (15.3)					
**Haematological Parameters**				**Haematological Parameters**			
Haemoglobin, gdL^−1^	10.5 (2.3)	10.1 (3.1)	**0.022**^**b**^	10.2 (2.2)	10.4 (1.6)	7.3 (1.3)	**0.001**^**c**^
Haematocrit, %	35.2 (6.8)	32.8 (9.0)	**0.009**^**b**^	34.6 (8.3)	32.8 (6.0)	26.4 (4.4)	**0.009**^**c**^
RBC, (× 10^12^ μL^−1^)	4.8 (0.7)	4.6 (1.3)	**0.045**^**b**^	4.6 (1.0)	4.8 (1.5)	3.2 (1.7)	**0.048**^**c**^
RDW, %	11.7 (3.0)	11.6 (2.8)	0.703^b^	11.3 (2.2)	11.5 (2.6)	14.9 (3.3)	**0.030**^**c**^
MCV, fL	76.3 (11.4)	75.2 (10.9)	0.349^b^	75.2 (9.9)	74.1 (11.8)	79.5 (15.9)	0.415^c^
MCH, fL/cell	22.7 (4.3)	22.5 (3.7)	0.744^b^	22.1 (3.7)	22.6 (4.0)	23.1 (3.5)	0.724^c^
MCHC, gdL^−1^	29.6 (3.7)	29.8 (3.0)	0.479^b^	29.7 (2.5)	30.9 (1.7)	27.7 (4.1)	0.054^c^
WBC (× 10^3^ μL^−1^)	8.5 (6.0)	8.6 (5.2)	0.746^b^	7.8 (4.8)	9.58 (5.2)	12.68 (4.6)	0.078^c^
Lymphocytes, (×10^3^ μL^−1^)	44.1 (25.3)	38 (66.9)	0.103^b^	36.55 (27.2)	37 (36.2)	50.9 (29.1)	0.596^c^
Monocytes, (×10^3^ μL^−1^)	9.3 (4.4)	11.2 (7.2)	0.084^b^	11.2 (7.4)	12 (7.8)	8.1 (6.1)	0.282^c^
Granulocytes, (×10^3^ μL^−1^)	46.1 (26.3)	50.0 (25.8)	0.354^b^	51.4 (27.2)	48.9 (27.5)	39 (28.2)	0.494^c^
Platelet Counts, (×10^3^ μL^−1^)	278.0 (112.8)	236 (129.5)	**0.001**^**b**^	220 (1127)	233 (129)	236(140)	0.399^c^
MPV, fL	5.5 (0.5)	5.40 (0.6)	0.119^b^	5.4 (0.6)	5.3 (0.9)	5.2 (0.8)	0.990^c^
PCT, %	0.2 (0.1)	0.13 (0.1)	**0.001**^**b**^	0.13 (0.1)	0.14 (0.1)	0.12 (0.1)	0.772^c^
PDW, %	9.4 (1.5)	9.5 (1.5)	0.629^b^	9.6 (1.4)	9.5 (2.0)	9.5 (0.40)	0.951^c^

Data are presented as the median (interquartile range; IQR) values unless stated otherwise. Study participants were categorized into non-infected and infected (with any density parasitaemia). ^a^ Statistical significance was determined by the Chi-square (χ^2^) analysis. ^b^ Statistical significance was determined using Mann- Whitney test. ^c^ Statisitical significance determined by the Kruskal-Wallis test

*Abbreviations*: *HbAA* Normal haemoglobin, *HbAS* Heterozygous sickle cell trait, *HbSS* Homozygous Haemoglobin S, *MCV* Mean corpuscular volume, *MCH* mean corpuscular haemoglobin, *MCHC* mean corpuscular haemoglobin concentration, *WBC* White blood cell count, *RBC* Red blood cells, *RDW* Red cell distribution width, *MPV* mean platelet volume, *PCT* Plateletcrit, *PDW* Platelet distribution width

### Haematological characteristics of *P. falciparum* infected children

Haematocrit levels were significantly higher in HbAA [median (IQR), 34.6 (8.3)] and HbAS [median (IQR), 32.8 (6.0)] relative to HbSS [median (IQR), 26.4 (4.4), *P* = 0.009]. Furthermore, red blood cell (RBC) count were equally significantly higher in HbAA [median (IQR), 4.6 (1.0)] and HbAS [median (IQR), 4.8 (1.5)] compared to HbSS [median (IQR), 3.2(1.7), *P* = 0.048]. Nonetheless, Red Cell Distribution Width (RDW) was relatively lower in HbAA [median (IQR), 11.3 (2.2)] and HbAS [median (IQR), 11.5 (2.6)] compared to HbSS [median (IQR), 14.9 (3.3), *P* = 0.030]. In cases where significant differences were detected using the Kruskal-Wallis test, we performed a post-hoc pairwise analysis using Dunn’s multiple comparison test. The post-hoc test was run on haemoglobin, haematocrit red blood cell count and red cell distribution width. Our results revealed that haemoglobin was reduced in children with HbSS than those with HbAS (*P* = 0.001). Furthermore, haematocrit was markedly reduced in children with HbSS than those with HbAS (*P* = 0.002). Red blood cell count was reduced in children with HbSS than those with HbAS (*P* = 0.023). However, red cell distribution width was highly raised in children with HbSS than those with HbAS (*P* = 0.001). There were no significant differences in the levels of other hematological indices and carriage of different *HBB* genotypes in children with *P. falciparum* malaria. These results are summarized in Table 1.

### Haematological abnormalities in carriage of different sickle cell genotypes in *P. falciparum* infection

To determine whether carriage of different sickle cell genotypes have effect on the severity of haematological abnormalities once an individual is infected, we evaluated their severity in children with *P. falciparum* infection and carriage of different sickle cell genotypes. Anaemia was defined according to the World Health Organisation’s criteria as a condition where haemoglobin level < 11 g/dl in children [23]. These severities were considered as described herein; based on haemoglobin level alone, severity of anaemia was categorised into groups of severe (haemoglobin level < 7 g/dl) mild (haemoglobin level 10–10.9 g/dl), moderate (haemoglobin level 7–9.9 g/dl) and non-anaemic (haemoglobin level > 11 g/dl) [13]. Severe anaemia was seen in 8.6% of children with HbAA, 7.1% with HbAS and 23.1% of those with HbSS. Moderate anaemia was seen in 34.5, 21.4 and 69.2% of HbAA, HbAS, and HbSS, respectively. Mild anaemia was detected in 22.4% in HbAA, 35.7% in HbAS and none in HbSS. Otherwise, non-anaemic children were also seen in 34.5, 37.5 and 7.7% in those with HbAA, HbAS and HbSS genotypes, respectively.

Thrombocytopenia was defined as a haematological condition in which platelet count is below 150 × 10^3^ μL^− 1^ of blood [24]. For evaluation purposes, severity of thrombocytopenia was categorized as severe (platelet count< 50 × 10^3^ μL^− 1^), moderate (platelet count between 50 and 100 × 10^3^ μL^− 1^) and mild (platelet count between 100 and 150 × 10^3^ μL^− 1^). Otherwise, any platelet count above 150 × 10^3^ μL^− 1^ was considered non-thrombocytopenic. None of the participants demonstrated severe thrombocytopenia in any of the *HBB* genotypes. Moderate thrombocytopenia was seen in 5.2 and 7.1% of HbAA and HbAS, respectively. However, none had thrombocytopenia in children with HbSS genotype. Mild thrombocytopenia was demonstrated in 15.5, 7.1 and 15.4% in individuals with HbAA, HbAS and HbSS, respectively. However, non-thrombocytopenia was demonstrated in 79.3% of children with HbAA, 85.7% of children with HbAS and 11% of children with HbSS.

Leucocytopenia was defined as a condition in which total leucocyte count is below 4 × 10^3^ μL^− 1^ of blood. There was no leucocytopenia in any of the study participants. Leukocytosis was defined as a benign abnormality in which total leucocyte count is above 11 × 10^3^ μL^− 1^) of blood [24]. Leukocytosis was demonstrated in 31% of children with HbAA, 42.9% in children with HbAS and in 69.2% of children with HbSS. Normal leucocyte count (WBC count between (4–11 × 10^3^ μL^− 1^) was seen in 70% of those with HbAA genotype, 57.1% in those with HbAS and in 30.8% in those with HbSS genotype.

Monocytosis was defined as a condition in which total monocyte count is below 12% of the total white blood cell count in peripheral blood [24, 25]. Monocytosis was seen in 59% in children with HbAA, 79% in children with HbAS and in 31% in children with HbSS genotype. The distribution of severity of anaemia, thrombocytopenia, leukocytosis, leucocytopaenia and monocytosis are summarized in Table 2.

**Table 2:** Distribution and severity of selected haematological abnormalities with regard to haemoglobin type in *P. falciparum* infected children

Haematological parameter	Severity of the Haematological Abnormalities	***HBB*** genotypes			Total n (%)
		HbAA n (%)	HbAS n (%)	HbSS n (%)	
**Haemoglobin**	Severe anaemia	5 (8.6)	1 (7.1)	3 (23.1)	9 (10.5)
	Moderate anaemia	20 (34.5)	3 (21.4)	9 (69.2)	32 (42.4)
	Mild anaemia	13 (22.4)	5 (35.7)	0 (0)	18 (21.2)
	Non-anaemic	20 (34.5)	5 (35.7)	1 (7.7)	25 (29.4)
	Total	58	14	13	85
**Platelets**	Severe thrombocytopenia	0 (0)	0 (0)	0 (0)	0 (0)
	Moderate thrombocytopenia	3 (5.2)	1 (7.1)	0 (0)	4 (4.7)
	Mild thrombocytopenia	9 (15.5)	1 (7.1)	2 (15.4)	12 (14.1)
	Non-thrombocytopenia	46 (79.3)	12 (85.7)	11 (84.6)	69 (81.2)
	Total	58	14	13	85
**Leukocytes**	Leukocytopaenia	0 (0)	0 (0)	0 (0)	0 (0)
	Leukocytosis	18 (31)	6 (42.9)	9 (69.2)	33 (38.8)
	Normal	40 (70)	8 (57.1)	4 (30.8)	52 (61.2)
	Total	58	14	13	85
**Monocytes**	Monocytosis	34 (59)	11(79)	4 (31)	49 (58)
	Normal	24 (41)	3 (21)	9 (69)	36 (42)
		58	14	13	85

Data are presented as proportions (n) and percentages (%) of different *HBB* genotypes and different severities of haematological abnormalitis in children infected with *P.falciparum* malaria

### Correlations between primary red cell measurements and red cell indices in children with carriage of different *HBB* genotypes (6GAG > 6GTG mutation)

Using partial correlation test while controlling for age and sex as factors that have previously been shown to affect haematological indices [26], the study revealed that in the HbSS genotype group, the RBC count had a negative correlation with RDW in children malaria-infected and non-infected (*r* = − 0.750, *P* = 0.008 and *r* = − 0.634, *P* = 0.049, respectively). Furthermore, there was a negative correlation between RBC count and both MCV and MCH in malaria-infected children (*r =* − 0.833, *P* = 0.001) and (*r* = − 0.750, *P* = 0.005), respectively. Haematocrit was negatively correlated with RDW in both malaria-infected and non-infected children (*r* = − 0.918, *P* = 0.001) and (*r* = − 0.718, *P* = 0.009), respectively. Additional analysis in the carriage HbAS genotype, showed a negative correlation between haemoglobin with RDW in malaria-uninfected children (*r* = − 0.694, *P* < 0.0001). The RBC count showed a negative correlation with MCV (*r* = − 0.752, *P* = 0.003) and with MCH (*r* = − 0.797, *P* = 0.001) in malaria-infected children.

In HbAA genotype, RBC count showed a negative correlation with MCV (*r* = − 0.324, *P* = 0.002), MCH (*r* = − 0.273, *P* = 0.010), MCHC (*r* = − 0.401, *P* = 0.000) and RDW (*r* = − 0.235, *P* = 0.027) in children without malaria infection. Haematocrit showed a positive correlation with MCV (*r* = 0.524, *P* < 0.0001), and a negative correlation with MCHC (*r* = − 0.333, *P* = 0.001) and RDW (*r* = − 0.361, *P* < 0.0001) in children without malaria infection. Haemoglobin showed a positive correlation with MCV (*r* = 0.525, *P* < 0.0001), MCH (*r* = 0.408, *P* < 0.0001) and MCHC (*r* = 0.269, *P* = 0.011), however, it showed a negative correlation with RDW (*r* = − 0.664, *P* < 0.0001) in children without malaria infection. The RBC showed a negative correlation with MCV (*r* = − 0.393, *P* = 0.003), MCH (*r* = − 0.408, *P* = 0.002), MCHC (*r* = − 0.270, *P* = 0.043) and RDW (*r* = − 0.399, *P* = 0.002) in malaria-infected children. There was a negative correlation between haematocrit and PDW (*r* = − 0.446, *P* = 0.001) in children infected with malaria. Haemoglobin showed positive correlation with MCH (*r* = 0.317, *P* = 0.016) and MCHC (*r* = 0.269, *P* = 0.011) in children infected with *P. falciparum* malaria. Further analysis revealed a negative correlation between haemoglobin and RDW (*r* = − 0.479, *P* = 0.001) in children infected with *P. falciparum* malaria and (*r* = − 0.479, *P* = 0.001) in children without *P. falciparum* malaria. It is therefore probable that the RBC count, haematocrit and haemoglobin concentration decrease with increase in RDW levels in *P. falciparum*-infected children with carriage of HbSS genotype relative to other *HBB* genotypes. Correlation between primary red cell measurements and red cell indices are summarized in Table 3.

**Table 3:** Correlation of haematological parameters with Sickle cell genotype in children with and without *P. falciparum* infection

			Malaria negative (***N*** = 132)				Malaria positive (***N*** = 85)			
Control Variable			MCV (fL)	MCH (Pg)	MCHC (gdL^−^)	RDW (%)	MCV (fL)	MCH (Pg)	MCHC (gdL^−^)	RDW (%)
**Sickle cell anaemia** (**HbSS) (*n*** **= 11)**							**Sickle cell anaemia (HbSS) (*n*** **= 13)**			
Age and sex	RBC count	r	−0.362	−0.068	0.060	−0.634	−0.883	−0.750	−0.118	−0.725
		*P* value	0.304	0.852	0.870	**0.049**	**0.001**	**0.005**	0.715	**0.008**
	Haematocrit	r	0.231	0.172	−0.086	− 0.918	− 0.555	− 0.418	0.273	− 0.718
		*P* value	0.521	0.635	0.813	**< 0.0001**	0.061	0.176	0.391	**0.009**
	Haemoglobin	r	−0.218	0.032	0.563	−0.616	− 0.526	− 0.220	0.521	− 0.792
		*P* value	0.545	0.929	0.090	0.058	0.079	0.493	0.082	**0.002**
**Sickle cell trait (HbAS) (*n*** **= 31)**							**Sickle cell trait (HbAS) (*n*** **= 14)**			
Age and sex	RBC count	r	−0.299	−0.494	−0.434	− 0.292	− 0.752	−0.797	− 0.305	−0.392
		*P* value	0.116	**0.006**	0.019	0.125	**0.003**	**0.001**	0.311	0.185
	Haematocrit	r	0.515	0.221	−0.458	−0.507	−0.269	−0.369	− 0.394	−0.506
		*P* value	**0.004**	0.249	**0.013**	**0.005**	0.375	0.214	0.183	0.078
	Haemoglobin	r	0.547	0.533	0.069	−0.694	−0.247	−0.235	−0.080	− 0.475
		*P* value	**0.002**	**0.003**	0.720	< **0.0001**	0.417	0.441	0.796	0.101
**Normal haemoglobin (HbAA) (*n*** **= 90)**							**Normal haemoglobin (HbAA) (*n*** **= 58)**			
Age and sex	RBC count	*r*	−0.324	−0.273	−0.401	− 0.235	− 0.393	−0.408	− 0.270	−0.399
		*P* value	**0.002**	**0.010**	**< 0.0001**	**0.027**	**0.003**	**0.002**	**0.043**	**0.002**
	Haematocrit	*r*	0.524	0.143	−0.333	−0.361	0.139	.076	−0.072	−0.446
		*P* value	**< 0.0001**	0.181	**0.001**	< **0.0001**	0.302	0.573	0.593	**0.001**
	Haemoglobin	*r*	0.525	0.408	0.269	−0.664	0.257	0.317	0.285	−0.479
		*P* value	< **0.0001**	< **0.0001**	**0.011**	< **0.0001**	0.053	**0.016**	**0.032**	**0.001**

Data are the partial correlations (*r*). Malaria negative patients (*n* = 132) and malaria positive patients (*n* = 85) with acute febrile illness were categorized on the basis of haemoglobin type. All statistical significance was determined by the partial correlation test (*r*) controlling for age and sex. Values in bold are statistically significant at *P* ≤ 0.05

*Abbreviations*: *MCV* Mean corpuscular volume, *MCH* mean corpuscular haemoglobin, *MCHC* mean corpuscular haemoglobin concentration, *RBC-Red blood cells, RDW* Red cell distribution width, *r* the measure of strength of Pearson’s correlation

## Discussion

Children from Sub-Saharan Africa continue to bear the largest burden of *P. falciparum* malaria [2]. Haematological changes or abnormalities are some of the most common complications in malaria and they play a major role in malaria pathogenesis [27, 28]. Previous studies have reported that haematological parameters are affected by *P. falciparum* infection and mutations in the *HBB* gene [19, 27, 29]. The presence of the sickle cell mutation has been shown to influence malaria outcome in populations where the infection is endemic [30] such as western Kenya. In the current study, we initially determined the allele frequency of the *HBB* rs334 in this population. Our results showed that the minor allele frequency (S) was 0.21, which is consistent with previous studies in other malaria endemic parts of Sub-Saharan Africa [31, 32]. The HWE inconsistency of the *HBB* rs334 allele distributions observed in the current study emphases that malaria infection is an important factor driving genetic drift towards sickle cell. In the current study, therefore, we determined severity of haematological abnormalities and correlation of haematological parameters with *HBB* genotypes in children with *P. falciparum* malaria in Kisumu County, western Kenya, a *P. falciparum* holoendemic region.

Our current study reveals the presence of severe anaemia in malaria-infected children with HbSS genotype. This observation could be due to chronic haemolysis observed in sickle cell anaemia [33] and accelerated sickling of infected RBCs culminating into ultimate clearance [34]. In addition, this could also be contributed by background haemolytic anaemia and autosplenectomy that occurs in children with HbSS [35, 36] and invasion of red cells of HbSS genotype by merozoites, which represents an external stressor that would make the cells to haemolyse before the parasites have a chance to reproduce [37]. Our finding that the hematocrit was lower in HbSS genotype was not surprising. The lower levels of haemoglobin contributes to low haematocrit percentage as a result of continuous haemolytic process in the carriage of HbSS, which then would lower the proportion of red blood cells in relation to the whole blood volume [26]. Our analysis also revealed that children with severe anaemia in HbSS genotype and infected with *P. falciparum* infection had increased red cell distribution width. The increased RDW in HbSS reported in the current study is explainable by pre-existing anisocytosis and poikilocytosis, which have been previously reported to be common biological conundrum in sickle cell disease [25, 26]. Increased RDW is further be attributed to existence of both mature normocytic cells and immature macrocytic red cell which have been prematurely released from the bone marrow in a compensatory response to chronic haemolysis in children with sickle cell [33]. The absence of anaemia in the smaller proportion of *P. falciparum*-infected children with HbSS genotype could be a modulated hereditary persistence of foetal haemoglobin (HPFH) [38]. It would be scientifically sound to explore this hypothesis in further studies.

The presence of mild and moderate anaemia was observed in children infected with *P. falciparum* and had the carriage of HbAA genotype. Even though the mechanism was not explored in the current study, this phenomena is attributable to reduced haemolytic mechanisms and slowed removal of infected red blood cells by erythro-phagocytosis in non-severe malaria [39]. Our findings demonstrate that the carriage of HbAA genotype is important in reducing anaemia severity. On the other hand, the observed few severe anaemia in the HbAA group could be due to increased haemolytic mechanisms and accelerated removal of infected red blood cells by erythro-phagocytosis and ineffective erythropoiesis [39]. The minimal severity of anaemia observed in the carriage of HbAS could be attributed to other anaemia-promoting conditions like poor nutritional status and infections such as HIV-1 and bacteremia [40], which the current study did not have the opportunity and ethical approvals to investigate. Other investigations have also implicated reduced anaemia (mild to moderate) in children with HbAS genotype relative to HbAA genotype and suggested that high levels of RBC complement regulatory proteins (CR1 and CD55) play a role in the pathogenesis of severe anaemia in malaria infection [41, 42]. Further, it is important to note that more children with the carriage of the HbAS genotype had mild to moderate anaemia and that children with carriage of the HbSS genotype and infected with *P. falciparum* infection had the lowest haemoglobin level as compared to HbAS and HbAA patients. This finding showed consistency with the findings of a similar study in hospitalized Cameroonian children with sickle cell anaemia and infected with malaria [43].

Even though lower platelet count was seen in the infected children relative to non-infected children in HbAA genotypes, it did not differ across other genotypes (HbSS vs. HbAS). This means that thrombocytopenia was not demonstrated in carriage of any haemoglobin type during *P. falciparum* infection. Our study did not find thrombocytopenia as a haematogical abnormality in children with *P. falciparum* malaria irrespective of the *HBB* genotypes. However, this finding differed from earlier studies which demonstrated thrombocytopenia in HbAA genotype individuals infected with *P. falciparum* [8, 9, 27]. This reported inconsistency in our study is attributable to the fact that study participants in the current study only suffered from non-severe malaria while in the previous study, the participants suffered from severe malaria. Previous studies have reported thrombocytopenia in *P. falciparum* infection and attributed this to peripheral destruction of platelets and their consumption by disseminated intravascular coagulation process, as well as splenic pooling [44]. On the other hand, thrombocytosis has been reported in HbSS individuals who are not infected with malaria [29]. Our finding needs further explorations since absence of thrombocytopenia and thrombocytosis which are known to be benign abnormalities of thrombocytes would not require any clinical intervention.

Our study further showed that children with HbSS genotype and infected with malaria had reduced monocytosis compared to those with HbAA and HbAS genotypes. A previous study showed a positive correlation between monocyte to lymphocyte ratio in the presence of malaria and the level of parasitaemia [45]. Furthermore, a previous study in the current study location demonstrated that monocytosis is the most important leucocytic change associated with *P. falciparum* malaria infection [27]. Monocytosis has been previously reported to drive sickle cell vaso-occlusion [17, 36] and it has also been reported to be associated with haemolysis and inflammation in sickle cell anaemia [18]. Monocytes are thought to be activated in sickle cell anaemia by the expression of TNF-alpha and IL-1 beta as well as the adhesion of molecule ligand (CD11b) [46–48]. Perhaps this could be an additional reason behind the utilization of hydroxyurea to alter the circulating monocyte subsets and to dampen its inflammatory potential in sickle patients apart from raising the level of fetal haemoglobin (HbF) [49]. In contrast, a previous study reported monocytopenia being associated with severe malaria and adverse outcome [44]. Initially, our finding revealed no significant difference in leucocyte count between malaria-infected versus non-infected children. Contrary to our finding, other studies have demonstrated leukocytosis [44, 50] while others have demonstrated leucocytopenia [51–53] in malaria-infected children with HbAA. Finding of the current study is comparable with other previous studies [27, 54, 55], which reported no significant differences between malaria-infected and non-infected children. Furthermore, leukocytosis was highest in the carriage of HbSS genotype in children infected with *P. falciparum* malaria. This finding was not surprising since it has been previously established that leukocytosis is a common biological response in severe malaria anaemia to counter the infection [56]. It is also important to point out that leucocyte adhesion and activation are major causes of sickle cell vaso-occlusion in sickle cell anaemia [57].

Mutations in haemoglobin beta sub-unit gene *(HBB)* have been found to primarily affect the red blood cells structurally resulting into sickle cell and physiologically affecting normal red cell functions [58]. However, it is not known how primary red cell measurement correlate with red cell indices in individuals infected with *P. falciparum* and presenting with different *HBB* (6GAG > 6GTG) genotypes. Erythrocyte counts revealed a strong negative correlation with RDW in children infected with malaria and had carriage of the HbSS genotype. Furthermore, this correlation trend was also seen in the malaria-infected children with the carriage of HbAS and HbAA. This implies that increasing the RDW reduces the erythrocyte counts once an individual is infected and this situation is aggravated by carriage of the HbSS genotype. These findings therefore demonstrate the effects of malaria infection on haematological parameters [59] and the effect of HbSS genotype on exacerbation of haematological abnormalities observed during infection.

Limitation in the current study included fewer number of subjects in HbAS and HbSS, lack of information on the nutritional status of the participants, possible presence of other co-infections e.g. HIV, bacteremia and intestinal infections such as hookworm and tapeworm which have been previously reported to worsen anaemia [40, 60, 61]. In addition, our study did not measure levels of fetal haemoglobin (HbF) as the most important biomarker for disease prognosis in SCD. The current study did not incorporate differential leucocyte count to give more insight to benign changes in cells of granulocytic series. Additionally, we lacked knowledge on the most recent malaria infectivity rate following several malaria control interventions in the study area and non-uniformity in the exposure of subjects to mosquitoes’ bites.

## Recommendation

We recommend that a similar longitudinal study with a larger sample size should be conducted in the same study area taking into account the level of HbF among other haematological parameters, as this may be more informative. We equally recommend inclusion of laboratory investigations of bacteremia and parasitic infections.

## Conclusion

Our study reveals that anaemia is the most common haematological abnormality in malaria-infected children with carriage of HbSS genotype. The RBC count, haematocrit and haemoglobin concentration decrease with increase in RDW levels in *P. falciparum*-infected children with carriage of HbSS genotype compared to other *HBB* genotypes. Therefore, carriage of HbSS genotype contributes to the severity of haematological abnormalities once a child is infected *P. falciparum*.

## Supplementary Information

**Additional file 1.**

## Data Availability

The datasets generated and/or analyzed during the current study are not publicly available due compliance with ethical reviewer guidelines but are obtainable from the corresponding author on reasonable request.

## Abbreviations

CR1: Complement regulatory protein 1
CD55: Cluster of differentiation 55
CD11b: Cluster of differentiation 11b
HbAA: Genotype of the normal haemoglobin
HbAS: Genotype in sickle cell trait
HbSS: Genotype in sickle cell anaemia
HWE: Hardy Weinberg Equilibrium
MCH: Mean corpuscular haemoglobin
MCHC: Mean corpuscular haemoglobin concentration
MCV: Mean corpuscular volume
PBF: Peripheral blood film examination
PCR: Polymerase Chain Reaction
RBC: Red cell count
RDW: Red cell distribution width
